# SARS CoV-2 Spike Protein *in silico* Interaction With ACE2 Receptors From Wild and Domestic Species

**DOI:** 10.3389/fgene.2021.571707

**Published:** 2021-02-15

**Authors:** Santiago Rendon-Marin, Marlen Martinez-Gutierrez, Gary R. Whittaker, Javier A. Jaimes, Julian Ruiz-Saenz

**Affiliations:** ^1^Grupo de Investigación en Ciencias Animales - GRICA, Facultad de Medicina Veterinaria y Zootecnia, Universidad Cooperativa de Colombia, Bucaramanga, Colombia; ^2^Infettare, Facultad de Medicina, Universidad Cooperativa de Colombia, Medellín, Colombia; ^3^Department of Microbiology and Immunology, College of Veterinary Medicine, Cornell University, Ithaca, NY, United States

**Keywords:** SARS-CoV-2, COVID-19, homology modeling, molecular docking, spike protein

## Abstract

The severe acute respiratory syndrome coronavirus 2 (SARS-CoV-2) has been declared a pandemic by the World Health Organization (WHO), and since its first report, it has become a major public health concern. SARS-CoV-2 is closely related to SARS-CoV and SARS-related bat coronaviruses, and it has been described to use angiotensin-converting enzyme 2 (ACE2) as a receptor. Natural SARS-CoV-2 infection in domestic and wildlife animals, measured by RT-qPCR, has been confirmed in different countries, especially from the Felidae family. *In silico* analysis of the interaction between the SARS-CoV-2 spike protein and the cellular receptor ACE2 in various animal species has suggested that wild felids and domestic cats could be susceptible to SARS-CoV-2 based on this interaction. Here, we performed a protein-protein molecular docking analysis of SARS-CoV-2 spike protein with the ACE2 receptor from different animals to elucidate the potential of those species as intermediate hosts or susceptible animals for SARS-CoV-2 infection. Compared to human ACE2, we found that ACE2 receptors from domestic cats and tigers could efficiently interact with RBD of SARS CoV-2 Spike protein. However, dog, ferret, and hamster ACE2 receptor interaction with SARS-CoV-2 S protein RBD was not predicted as favorable, demonstrating a potential differentiated susceptibility in the evaluated species.

## Introduction

A novel coronavirus (CoV) named severe respiratory syndrome coronavirus-2 (SARS-CoV-2) emerged in China in December 2019 (ICTV, 2020). This virus has been confirmed as the causative agent of the Coronavirus disease 2019 (COVID-19) which has now grown into a pandemic, declared by the World Health Organization (WHO) on March 3rd, 2020 (WHO, 2020). Multiple phylogenetic studies have supported the hypothesis that SARS-CoV-2 could originate in animals, specifically in wild populations of bats in Asia (Jaimes et al., 2020a; Li et al., 2020c; Zhou et al., 2020). As with other betacoronaviruses, SARS-CoV-2 is an enveloped virus and its genome is composed of a single-stranded, non-segmented, positive sense RNA molecule (Masters and Perlman, 2013). The virus belongs to the *Orthocoronavirinae* subfamily, *Coronaviridae* family, order Nidovirales (ICTV, 2018). The *Orthocoronavirinae* encloses four genera: Alpha-, Beta-, Delta-, and Gamma-CoV. Alpha- and Beta-CoV are reported to have originated in mammals, and Delta- and Gamma-CoV are reported to have an origin in avian viruses (Woo et al., 2012). While Delta- and Gamma-CoVs are known to infect birds and nonhuman mammals, both Alpha- and Beta-CoV have been reported to have the ability of being transmitted from animals to humans (Cui et al., 2019).

SARS-CoV-2 has been reported to be closely related to the Severe Acute Respiratory Syndrome coronavirus (SARS-CoV) first described in 2002 (Al-Tawfiq et al., 2014), where transmission was initially linked to the Himalayan palm civets (*Paguma larvata*) and the raccoon dogs (*Nyctereutes procyonoides*; Xu et al., 2009; Cui et al., 2019). During the 2002–2003 SARS-CoV emergency, the presence of the virus was also reported in cats, coinciding with places where SARS-CoV-positive humans lived, and with some of them displaying signs of disease (Wang et al., 2005). Moreover, experimental infection in cats and ferrets has demonstrated the susceptibility of these species to SARS-CoV, evidenced by the infection spreading to multiple organs, viral excretion, and transmissibility to susceptible contacts (Martina et al., 2003). It has been also described that subclinically infected cats show similar pathological lesions to those found in humans infected with SARS-CoV, characterized by the presence of diffuse alveolar damage, and especially in cats, tracheobronchoadenitis (van den Brand et al., 2008).

SARS-CoV-2 originated in Wuhan City, in the Hubei Province, and it has been suggested it may have originated in a wet market where more than 120 live animal species were found, consistent with the idea that zoonotic infections occur where people are in contact with or consume different animals (Chan et al., 2020a; Chen et al., 2020; Huang et al., 2020; Li et al., 2020b; Zhu et al., 2020). During the current SARS-CoV-2 pandemic, several reports have suggested the possible infection of wild and domestic animals with this virus (CDC, 2020; Daly, 2020; Sit et al., 2020; Zhang et al., 2020). In addition, three studies have described the SARS-CoV-2 infection in several domestic species under experimental conditions (Halfmann et al., 2020; Kim et al., 2020; Shi et al., 2020). These reports have generated international attention and rising concerns about the role of animals in SARS-CoV-2 epidemiology and its possible role in viral transmission (Bonilla-Aldana et al., 2020; Gollakner and Capua, 2020; Tiwari et al., 2020). Natural infection in domestic animals, measured by RT-qPCR, has been confirmed in different countries by the World Organization for Animal Health (OIE). In Hong Kong, dogs (Sit et al., 2020) and cats were found positive; in Belgium, Spain, Germany, and France, different domestic cats have been found positive, most of them being housemates of COVID-19-positive humans (OIE, 2020). Recently, two domestic cats and several wild felids (tigers and lions) have tested positive for SARS-CoV-2 at the Bronx Zoo in New York City (CDC, 2020; Daly, 2020). In contrast, serological evidence has found the presence of SARS-CoV-2-specific antibodies in 14.7% if the surveyed cats in Wuhan as indicative of exposure and possible infection with the virus (Zhang et al., 2020). Moreover, the Animal and Plant Health Inspection Service agency has confirmed SARS-Cov-2-positive Snow Leopard at a Louisville Zoo, in Kentucky, by RT-qPCR after animals exhibited signs of respiratory illness (USDA, 2020).

Experimental *in vivo* inoculation of SARS-CoV-2 has shown no infection in dogs, pigs, chickens, and ducks, suggesting that these species are not susceptible to the virus (Shi et al., 2020). However, the same study showed that intranasally infected cats and ferrets resulted in positive infection and possible transmission to other susceptible animals through respiratory droplets (Shi et al., 2020). More recently, an experimental infection in cats confirmed the presence of both viral RNA, and infectious SARS-CoV-2 viral particles in nasal swabs of infected cats and close contact housed animals. Interestingly, none of the cats (infected and contacts) showed any clinical symptoms, and no virus nor viral RNA was detected in rectal swabs. However, the IgG antibody titers ranked between 1/5120 and 1/20,480 on day 24 after the initial inoculation, suggesting an active immune response against the virus (Halfmann et al., 2020).

In ferrets, two independent studies have shown that SARS-CoV-2 could be efficiently transmitted by direct contact and aerosols (Kim et al., 2020; Richard et al., 2020). SARS-CoV-2-infected ferrets exhibited elevated body temperatures and virus replication. Although fatalities were not observed, viral shedding in nasal washes, saliva, urine, and feces was detected up to 8 days postinfection. Furthermore, naïve contact ferrets were also found positive for viral RNA or virus detection in both studies, suggesting an efficient airborne transmission between animals of this species (Kim et al., 2020; Richard et al., 2020). Moreover, in minks, diverse studies and reports have exhibited their susceptibility to SARS-CoV-2 both, naturally and experimentally (Johansen et al., 2020; Oreshkova et al., 2020; Janik et al., 2021). Whole-genome sequencing on mink farms and farm workers was executed, finding that SARS-CoV-2 was introduced from humans and likely showed a widespread circulation among mink in the beginning of the infection period some weeks before it was detected (Koopmans, 2020; Munnink et al., 2020).

Besides, the hamster model has been widely employed for a medical study through nasal SARS-CoV-2 inoculation, in which not only viral replication and lung inflammation were observed in infected animals but also infection were observed in co-housed animals (Chan et al., 2020c; Cleary et al., 2020). Furthermore, the replicative ability of SARS-CoV-2 in different organs in two different age groups of hamsters after inoculation by the nasal and ocular routes was assayed, indicating that the virus could replicate efficiently in the respiratory tracts of both young and older infected animals (Imai et al., 2020), suggesting hamsters as a suitable animal model for further studies of SARS-CoV-2 pathogenesis and transmission, since Golden Syrian hamster was employed as an experimental animal model and was reported to support replication of SARS-CoV (Sia et al., 2020).

The angiotensin-converting enzyme 2 (ACE2) serves as a functional receptor for the spike protein of SARS-CoV and SARS-CoV-2 (Li et al., 2005; Lan et al., 2020). This enzyme is the key element in the renin-angiotensin system, with a crucial role in the regulation of blood pressure in vertebrates (Lv et al., 2018). In humans, ACE2 could be polymorphic and although mRNA is known to be present in virtually all organs, the protein expression is limited to certain tissues including small intestine, testis, kidneys, heart, thyroid, lungs, colon, liver, bladder (Li et al., 2020a). Moreover, this cellular receptor could be present in arterial and venous endothelial cells and arterial smooth muscle cells (Hamming et al., 2004), which might provide some clues for understanding the atypical pathogenesis of the COVID-19.

In animals, differential expression of ACE2 in tissues has not been widely studied to date. However, it has been recently reported that the ACE2 gene is highly conserved among common mammals at both the DNA and protein levels, suggesting that SARS-CoV-2 can potentially bind to different ACE2 orthologous proteins from mammals (Conceicao et al., 2020; Sun et al., 2020). In domestic cats and dogs, the ACE2 gene is highly expressed in various tissues such as kidney, heart, and liver. In cats, ACE2 has been demonstrated to be highly expressed in skin, ear tip, lungs, and retina, and in dogs, ACE2 is expressed in skin and retina (Sun et al., 2020). Furthermore, the ACE2 expression in the lungs of cats and ferrets has been observed, which could suggest that these animals may be more suitable for SARS CoV-2 studies than the rodent models (Guo et al., 2008; Sun et al., 2020).

Supporting the *in vivo* experimental studies, recent *in silico* analyses of the interaction between the SARS-CoV-2 spike protein and the cellular receptor ACE2 in various animal species have suggested that wild felids and domestic cats could be susceptible to SARS-CoV-2 (Damas et al., 2020). The role of cats in the transmission cycle of SARS-CoV-2 is still unknown; however, considering that the interaction between the viral and the cellular receptor has been shown to be similar to that observed in humans (Wu et al., 2020), it is crucial to determine if cats, as well as other animals, could act as a reservoir or intermediary host for the virus. Here, we performed a protein-protein molecular docking analysis of SARS-CoV-2 spike protein with the ACE2 receptor from dogs, cats, tigers, ferrets, and hamsters to better understand the potential of those species as intermediate hosts or susceptible animals for SARS-CoV-2 infection.

## Materials and Methods

### ACE2 Sequences and Multiple Alignments

Amino acid sequences of the cat, tiger, dog, hamster, and ferret ACE2 receptors were retrieved from GenBank (accession numbers: AAX59005.1, XP_007090142.1, NP_001158732.1, XP_003503283.1, and BAE53380.1, respectively). A multiple-sequence alignment was performed to evaluate the amino acid identity among ACE2 receptors compared to the human ACE2 (NP_001358344.1). The p-distance matrix was calculated, and the ACE2-virus-binding interface interacting with SARS-CoV-2 Spike protein RBD was also aligned in order to determine the amino acid substitutions among the selected species.

### Homology Modeling and Model Validation

Homology models of the ACE2 cellular receptors of cats, tigers, dogs, hamsters, and ferrets were built to study their interaction with RBD of SARS-CoV-2. A co-crystal of RBD SARS CoV-2 Spike protein and human ACE2 was obtained from Protein Data Bank (PDB# 6M17). Human ACE2 was used as templated, and MODELLER v. 9.24 was employed to obtain the 3D structure models of cat, tiger, ferret, and hamster ACE2 cellular receptors. After running the simulation, 100 different structures with different model quality scores (molpdf, DOPE, GA341; Sali and Blundell, 1993) were constructed. The homology models of the animal ACE2 cellular receptor and the human ACE2 templates were structurally aligned to assay the root mean square deviation (RMSD) differences among the model and template structures through TM-Align, a protein structure alignment algorithm based on the TM score (Zhang and Skolnick, 2005). The homology models were evaluated with computational tools as SWISS-MODEL™, which calculates the Z-score, QMEAN, and Ramachandran plot (Benkert et al., 2011). ProSA-Web, another Z-score for the overall model quality, enables to establish whether the z-score value of the model structure is located in the range of Z-scores exhibited by native proteins of similar size, with PDB as the reference database (Wiederstein and Sippl, 2007). All these computational tools enable to determine whether 3D models of animal ACE2 cellular receptors are reliable models to employ in molecular docking analysis.

### Molecular Docking Analysis

Fully glycosylated (Woo et al., 2020) and non-glycosylated RBD of SARS CoV-2 Spike protein and the modeled animal ACE2 cellular receptors were docked employing a molecular docking online tool, HADDOCK 2.4 (van Zundert et al., 2016), a flexible docking approach, in order to elucidate whether animal cellular receptors could interact with RBD SARS CoV-2 Spike protein, and its interaction with the human ACE2 cellular receptor was used as control; this complex was also submitted to the molecular docking analysis (redocking). For all the molecular docking tools, the ligand and the receptor are considered of rigid structure. A total of 200 and 400 simulations were carried out for either non-glycosylated or glycosylated RBD of SARS CoV-2 Spike protein. All 3D complex graphics were generated using the software UCSF Chimera (Pettersen et al., 2004). The cACE2 crystallographic structure was released (PDB# 7C8D) in the complex to RBD of SARS-CoV-2; at the same time, this work was carried out. A structural alignment was employed to determine whether the post dock result from cACE2 and the SARS CoV-2-RBD complex is comparable to the crystallographic structure.

## Results

### ACE2 Amino Acid Identity Among the Studied Species

ACE2 cellular receptors orthologs are expressed in several cell types of most animal species. In order to study the ACE2 identity among cats, tigers, hamsters, dogs, ferrets, and humans, a multiple-amino acid sequence alignment was performed. As shown in Table 1, the human ACE2 (hACE2) differs by only 14.2, 13.8, and 16.0% with the cat (cACE2), tiger (tACE2), and dog (dACE2) orthologs, respectively. Moreover, hACE2 differs in 15 and 17.2% with hamster (haACE2) and ferret (fACE2) orthologs (Table 1).

**Table 1:** *p*-distance matrix of cat, tiger, dog hamster, ferret, and human ACE2 cellular receptor sequences.

		hACE2	cACE2	tACE2	fACE2	dACE2	haACE2
1	hACE2						
2	cACE2	0.142					
3	tACE2	0.138	0.011				
4	fACE2	0.172	0.104	0.106			
5	dACE2	0.160	0.092	0.094	0.104		
6	haACE2	0.150	0.158	0.160	0.166	0.166	

**Figure 1:**
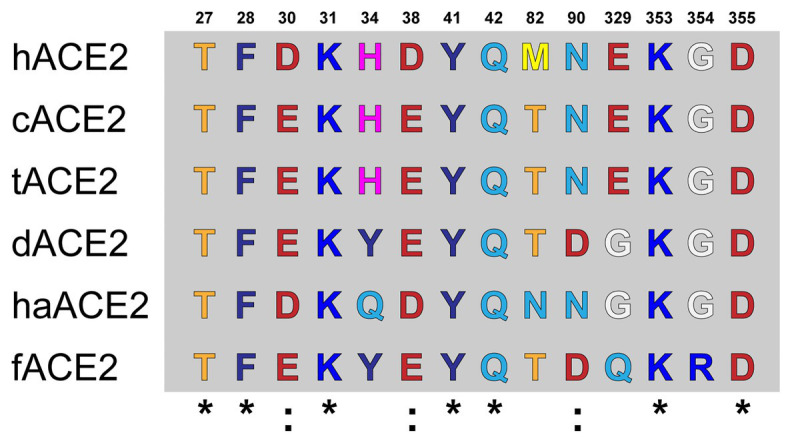
Amino acid alignment of the ACE2-virus-binding interfaces. Comparative analysis of ACE2 amino acid composition in the ACE2-virus-binding interface. The ACE2 sequences of fACE2, tACE2, haACE2, dACE2, fACE2, and hACE2 were aligned, and the amino acids found at the residue positions in the binding interface are shown. Conserved residues compared to hACE2 are noted (^*^).

Amino acid differences were observed at the ACE2-virus-binding interface of the studied species (Figure 1). cACE2- and tACE2-virus-binding interfaces showed the higher identity to hACE2. Both orthologs shared the same amino acid sequence but were different to the hACE-2-virus-binding interface in three residues: D30E, D38E, and M82T (Figure 1). Similarly, haACE2 showed three substitutions at the positions: H34Q, M82N, and E329G compared to the human ortholog (Figure 1). In contrast, dACE2- and fACE2-virus-binding regions possessed a larger number of amino acid substitutions compared to hACE2. dACE2 has six amino acid substitutions at positions D30E, H34Y, D38E, M82T, N90D, and E329G, while fACE2 displayed seven substitutions at positions: D30E, H34Y, D38E, M82T, N90D, E329G, and G354R (Figure 1).

### ACE2 Receptor Homology Modeling

**Figure 2:**
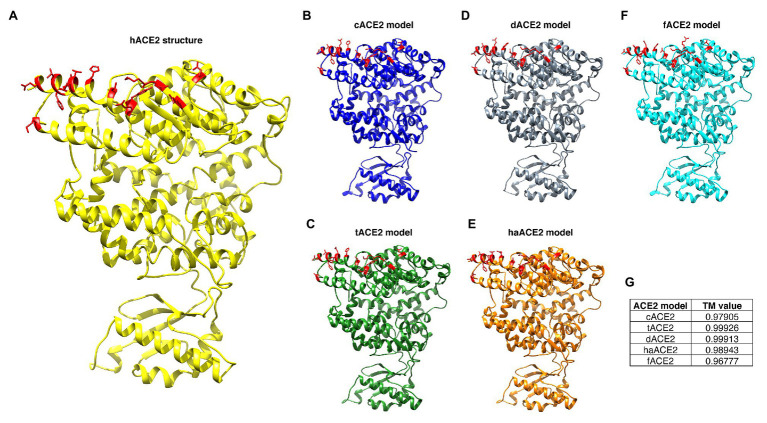
ACE2 homology models of selected species. hAEC2 structure (PDB# 6M17; **A**). Homology models of cACE2 **(B)**, tACE2 **(C)**, dACE2 **(D)**, haACE2 **(E)**, and fACE2 **(F)** were built based on the hACE2 structure. ACE2-virus-binding interfaces are marked in red. **(G)** TM values for the homology models are noted.

Amino acid sequences for the cat, tiger, hamster, dog, and ferret ACE2 receptors were used for building homology models based on the structure of the hACE2 receptor (PDB# 6M17; Figure 2). Models were validated by computational analysis, and all had more than 95% of the amino acids in favorable regions for rotations and folding (Table 2). The overall quality of the models was evaluated using ProSA Web. All models were located within the distribution of X-ray crystallography protein structures (Table 2). The folding homology of the models was also evaluated using the TM parameter. All models displayed TM values above 0.96 (Table 2). Overall, our predicted models met the quality requirements for homology with the hACE2 structure.

**Table 2:** Validation data for the predicted ACE2 homology models.

Protein	Z value	Favorable region (%)	TM value	Align AA
cACE2	−13.8	96.6	0.97905	746
tACE2	−13.7	96.5	0.99926	747
dACE2	−13.3	96.7	0.99913	747
haACE2	−13.1	96.7	0.98943	747
fACE2	−13.6	96.7	0.96777	747

### SARS-CoV-2-ACE2 Receptor Interaction Prediction

To evaluate whether SARS CoV-2 Spike protein could interact with ACE2 cellular receptors from the selected species, molecular docking tools were employed and the co-crystal structure of SARS-CoV-2 Spike RBD and human ACE2 cellular receptor interaction (PDB# 6M17) was used as control for interface interaction analyses. To assess the reproducibility of our molecular docking analyses, the SARS-CoV-2 Spike RBD and the hACE2 cellular receptor structures were subjected to re-docking analysis and the results agreed with those reported in the co-crystal structure (data not shown). The molecular docking site for the predicted ACE2 homology models was determined based on the amino acids mapping the binding interfaces of SARS-CoV-2 Spike RBD-hACE2 cellular receptor co-crystal structures (PDB# 6M17). Protein-protein interaction complexes were evaluated using the HADDOCK 2.4 server. This computational tool allows the simulation of the molecular interaction between proteins, and the clustering analysis implies ligand flexibility. The top 10 clusters were analyzed, and the best scores from each cluster were compared to of SARS-CoV-2 Spike RBD-hACE2 cellular receptor co-crystal structures (PDB# 6M17). Simulations were also carried out with SARS-CoV-2 Spike glycosylated RBD (Table 3).

**Table 3:** Molecular docking and clustering score ACE2 homology models in complex with SARS CoV-2 Spike protein RBD.

ACE2 in complex	S protein modifications	Cluster number	Cluster size^a^	HADDOCK energy score^b^	Overall RMSD (Å)^c^	Z-score^d^
cACE2	Non-gly	1	183	−123.9 ± 1.9	0.8 ± 0.5	−1.0
	Gly	1	400	−138.1 ± 3.4	0.5 ± 0.3	0.0
tACE2	Non-gly	1	165	−136.7 ± 1.2	1.3 ± 0.9	−1.8
	Gly	1	380	−137.6 ± 0.9	0.5 ± 0.3	−1.0
dACE2	Non-gly	4	7	−127.5 ± 21.3	2.1 ± 1.2	−0.5
	Gly	2	6	−123.5 ± 2.3	3.1 ± 1.2	−1.5
haACE2	Non-gly	2	60	−106.4 ± 3.4	15.4 ± 0.4	−0.2
	Gly	2	23	−120.5 ± 2.9	17.7 ± 0.1	1.0
fACE2	Non-gly	2	4	−120.6 ± 4.0	1.2 ± 0.5	1.0
	Gly	1	400	−138.1 ± 2.9	0.8 ± 0.7	0.0
hACE2	Non-gly	1	181	−126.1 ± 1.4	0.6 ± 0.4	−1.3
	Gly	1	388	−133.4 ± 2.5	0.7 ± 0.5	−1.0

a Based on a total of 200 generated models.

b Best HADDOCK score clusters are reported based on the lowest energy score, calculated as the sum of Van der Waals, electrostatic, desolvatation, and restraint energies, as an average of the four best structures of the cluster.

c RMSD among the best four structures of the cluster.

d Z-Score calculated as the standard deviations of the HADDOCK score of the cluster separated from the mean of all clusters.

**Figure 3:**
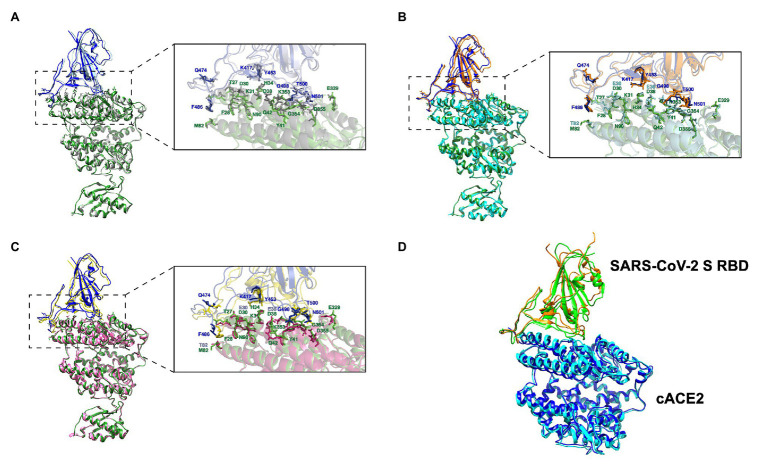
Homology docking analysis hACE2-, cACE2-, and tACE2-SARS-CoV-2 Spike protein RBD complexes. **(A)** hACE2 (gray) and SARS-CoV-2 Spike protein RBD (light blue) complex. **(B)** cACE2 (cyan) and SARS-CoV-2 Spike protein RBD (orange). **(C)** tACE2 (pink) and SARS-CoV-2 Spike protein RBD (yellow). Binding interface residues are noted (sticks). hACE2 and SARS-CoV-2 Spike protein RBD structures (PDB# 6M17) and interacting residues are colored in green and blue, respectively. Amino acid substitutions for cACE2 and tACE2, compared with hACE2, are shown in cyan and pink, respectively. **(D)** Structural alignment of cACE2 (cyan) and SARS-CoV-2 Spike protein RBD (orange) from the cACE2-RBD complex obtained by molecular docking and the cACE2-RBD (blue and green, respectively) complex from the crystallographic structure (PDB# 7C8D).

Molecular docking simulation of the hACE2 and the SARS CoV-2 Spike RBD was used as positive control (HADDOCK score: −126.1 ± 1.4) and compared to PDB# 6M17 (Figure 3A). We performed the same docking simulation for cACE2 and tACE2, which were the orthologs that showed higher scores in the HADDOCK energy analysis (Table 3) when a non-glycosylated SARS CoV-2 Spike RBD was used. The docking simulation for cACE2-RBD (HADDOCK score of −123.9 ± 1.9) showed high conservation in the ACE2-virus-binding interface, and a similar interaction with SARS-CoV-2 RBD (Figure 3B), suggesting that SARS-CoV-2 could establish stable interactions with cACE2. Similar results were observed in the tACE2-RBD docking simulation (Figure 3C). Interestingly, tACE2-RBD scored −136.7 ± 1.2 in the HADDOCK analysis. This value is lower than the predicted hCE2-RBD interaction (Table 3), which suggests that the tACE2-SARS-CoV-2 RBD bound could be stronger and more stable, which could contribute to SARS-CoV-2 entry in tiger cells. Moreover, when the glycosylated SARS CoV-2 Spike RBD was employed, for cACE2 and tACE2, cluster number and docking scores were comparable, indicating that glycosylation has not implied changes in the docking analysis (Table 3). It is noteworthy, for fACE2 simulations, that the enlargement of simulations (400 simulations instead 200) and glycosylation consideration have an effect in cluster number, size, and docking score, suggesting an important contribution of those abovementioned features (Table 3). It is important to mention that, as has been reported recently (Woo et al., 2020), no glycosylation was present through the interaction region of SARS CoV-2 Spike RBD with the ACE2 cellular receptor.

Neither dACE2- nor haACE2-RBD docking simulation analyses showed comparable interaction to the co-crystal hACE2-SARS-CoV-2 Spike protein RBD in the most probable cluster for either non-glycosylated or glycosylated SARS-CoV-2 Spike RBD. Moreover, clustering analysis disfavor these complexes, suggesting that interaction between SARS-CoV-2 and dACE2 and haACE2 is not as stable as in with hACE2, cACE2, and tACE2.

At the time of the manuscript construction, the cACE2 crystallographic structure was elucidated (PDB# 7C8D). A structural alignment was done, and TM-Align of 0.96690 and RMSD value of 1.5 Å indicated that both structure complexes have identical folding (Figure 3D). Thus, the molecular docking simulation could reproduce experimental data, not only for the hACE2-RBD complex but also for the cACE2-RBD complex, suggesting the reproducibly of our molecular docking analyses in comparison to experimental data.

## Discussion

SARS-CoV-2 has rapidly expanded into the human population causing more than 76.2 million cases, and COVID-19 had claimed almost 1,699,230 lives worldwide as of December 22, 2020 (WHO, 2020). It has been suggested that intermittent distancing as a measure for control may be required into 2022, unless critical care capacity is increased substantially or a treatment or vaccine becomes widely available (Kissler et al., 2020). Alongside human cases, multiple cases of human-to-animal transmission have emerged, including cases in endangered captive species (CDC, 2020; Daly, 2020; OIE, 2020; Sit et al., 2020), suggesting that animals could be susceptible to SARS-CoV-2 and rising questions about their role in the virus transmission.

Using computational tools, we first studied the amino acid identity between the cACE2, tACE2, dACE2, haACE2, and fACE2 receptors and compared them to hACE2. A high conservation was observed between the studied ACE2 receptors at the amino acid sequence level (Table 1). A high amino acid sequence identity of the ACE2 receptor has been observed in several species (Lam et al., 2020), suggesting that the gene is highly conserved among mammals. At the ACE2-virus-binding interface of the selected species, we observed a high amino acid identity at the receptor-virus-interacting residues (Figure 1) and agreeing with previous reports (Lam et al., 2020; Stout et al., 2020). Based on the amino acid sequence results, we wanted to study the structural organization of these orthologs in comparison to hACE2. Unfortunately, the structure of most animal ACE2 receptors has not been solved yet, limiting the study of these proteins.

Homology modeling is a helpful approach that predicts the 3D structure of protein using a known protein structure (template structure), and it is an accurate computational method for protein structure determination and prediction (Vyas et al., 2012). We have previously used this approach to predict the structural organization of viral proteins (including SARS-CoV-2 Spike), and we have tested the accuracy and potential of this tool for structural analyses (Jaimes and Whittaker, 2018; Jaimes et al., 2020a,b; Tang et al., 2020). Using this tool, we built and validated homology models for the selected ACE2 orthologs based on the hACE2 structure (PDB# 6M17; Figure 2). The model accuracy was validated, and all the predicted models had a TM value above 0.96 (Table 2). We observed that the overall organization of the ACE2 receptor of the selected studied species remarkably resembles the hACE2 structure (Figure 2), suggesting that these orthologs probably undergo similar molecular processes and could potentially interact with the SARS-CoV-2 receptor (i.e., Spike protein RDB).

To better study the ACE2-SARS-CoV-2 receptor interaction, we developed homology models based on the hACE2-SARS-CoV-2 Spike protein RBD structure (PDB# 6M17; Figure 3). We validated the models for protein-protein docking interaction using the HADDOCK 2.4 server (Table 3). Our analysis predicted that the cACE2 and tACE2 receptor could potentially interact with the RBD of SARS CoV-2 Spike protein with similar strength as it was previously reported for hACE2 (Damas et al., 2020; Wu et al., 2020). These results suggest that both cat and tiger ACE2 receptors could potentially establish stable interactions with SARS-CoV-2 Spike protein RBD. Several reports have raised the questions on whether or not domestic and wild species (specially felids) could be susceptible to SARS-CoV-2 infections (Bonilla-Aldana et al., 2020; Lam et al., 2020; Stout et al., 2020). In addition, laboratory virus-challenge studies have shown that cats can get infected with SARS-CoV-2 after inoculation with high amounts of virus (Bosco-Lauth et al., 2020; Halfmann et al., 2020; Shi et al., 2020). However, none of these studies have provided evidence of the susceptibility of these species at the cellular receptor level, leaving a gap in the understanding of the molecular processes behind this susceptibility. While our study is based on homology models, the predicted results strongly suggest that cACE2 and tACE2 can efficiently interact with SARS-CoV-2, supporting the hypothesis about the susceptibility of these species to the infection.

Ferrets and hamsters have both been efficiently experimentally infected, showing signs of diseases and transmitting the virus directly and through the air (Chan et al., 2020b; Richard et al., 2020; Shi et al., 2020), and have been employed as a suitable animal model to understand the main aspects of SARS-CoV-2 pathophysiology, pathogenesis, and transmission (Johansen et al., 2020). Moreover, natural infection of minks was widely confirmed (Halfmann et al., 2020) with remarkable importance in public health, since animal-to-human transmission of SARS-CoV-2 within mink farms was confirmed (Koopmans, 2020; Munnink et al., 2020).

Our results are in the same context as others, indicating a low ACE2-binding score in SARS-CoV-2 Spike non-glycosylated RBD (Cao et al., 2020; Damas et al., 2020; Lam et al., 2020) for ferrets and hamsters. This discrepancy could be related to the high infectivity dose used for experimental infection which likely does not correspond to virus exposure in nature (Damas et al., 2020) or the presence of different cellular factors that even, in low interaction with cellular ACE2 receptors, lead to highly effective replication in respiratory tissues (Kim et al., 2020; Richard et al., 2020; Shi et al., 2020). Diverse genetic and structural analyses have elucidated potential interaction models of SARS-CoV-2 S protein and orthologs ACE2 classifying fACE2 and hamACE2 with low affinity for SARS-CoV-2 S protein (Brooke and Prischi, 2020; Luan et al., 2020; Zhai et al., 2020).

Multiple studies deposited in preprint servers have proposed SARS-CoV-2 ACE2-independent binding to glycosaminoglycans, heparan sulfate, and heparin (Chiodo et al., 2020; Hao et al., 2020; Liu et al., 2020; Mycroft-West et al., 2020; Partridge et al., 2020). This mechanism has been also described in other coronaviruses (Millet et al., 2020) and should be further studied in the context of SARS-CoV-2 infection in different species. These studies are also highly relevant for understanding of the SARS-CoV-2 infection in ferrets and hamsters.

The analysis of different substitutions at binding sites of the orthologous ACE2 cellular receptor enables to understand the possible susceptibility and propose a molecular explanation for the interaction between SARS-CoV-2 and different species. The reduced experimental evidence recently reported to date suggests that while cats – which have the same residue as humans at sites 34 and 90 – are not strongly symptomatic and present lung lesions, while dogs – which have a substitution at this site – do not (Shi et al., 2020). Cats may be readily infected though, at a subclinical level.

We need to consider that a very reduced number of sequences are available for felid analysis, while for nonhuman primates there are at least 19 different primates to evaluate and make conclusions (Damas et al., 2020; Melin et al., 2020); there are only few different available sequences belonging to the domestic cat and felids that could be used, and the possibility to extrapolate this results to other felids is not plausible. However, recent confirmation of infection in captive lions in the New York Zoo (USDA, 2020) and unpublished results from Barcelona Zoo, where four lions tested positive for COVID-19, confirmed the risk for most of the big cats in captivity and close contact to COVID-19-positive human subjects.

It has been recently stated that species carrying a sequence with K31, Y41, N90, and K353 in ACE2 could be classified as highly susceptible to infection by SARS-CoV-2 (including human, nonhuman primates, and bats) while others should be less susceptible to infection except if the virus adapts to a second receptor for cellular binding and entry (Devaux et al., 2020). Our results strongly support this hypothesis in tigers and domestic cats (Figure 1). Moreover, we confirmed that those felids have comparable interaction interfaces in comparison to co-crystal human ACE2 and RBD SARS CoV-2 Spike protein (Figure 3).

Our results are also consistent with the earlier observation on SARS-CoV (Li et al., 2005) and the recent crystallographic analyses on SARS CoV-2 Spike protein (Yan et al., 2020) binding to ACE2, indicating that N90 was highly relevant for the attachment of the RBD of Spike protein on both viruses. In this context, it is important to denote that in both pandemics, natural and experimental infection of domestic cats was reported (Martina et al., 2003; Shi et al., 2020; Zhang et al., 2020). However, although our results support the hypothesis of susceptibility of infection, there is no current information about the disease and transmission of SARS-CoV-2 to other susceptible animals.

Tissue distribution of ACE2 in humans has been highly correlated with clinical manifestations of COVID-19 (Cao et al., 2020; Li et al., 2020a; Xu et al., 2020), including a possible explanation for atypical clinical manifestations of COVID-19 and different excretion ways (Li et al., 2005, 2020a). A high expression of ACE2 in type II alveolar cells enables a fast viral colonization, leading to a local alveolar wall destruction, with severe diffuse alveolar damage, implying migration of immune cells through the blood-air barrier contributing to complications of COVID-19 as venous thromboembolic disease and multiple-organ involvement (Huertas et al., 2020; Ortiz et al., 2020; Ziegler et al., 2020). Nevertheless, a very few datasets could help to understand the differential expression of ACE2 in feline tissues. It has been recently reported for domestic cats that ACE2 was highly expressed in kidney, skin, ear tip, pancreas, and liver and in a minor proportion in lungs and muscle (Sun et al., 2020). Those results are partially correlated with the recent experimental infection of cats, which found low viral replication in intestine and other tissues (brains, hearts, submaxillary lymph nodes, kidneys, spleens, livers, and pancreas), in comparison to lungs, trachea, and nasal turbinate (Shi et al., 2020). Nonetheless, although natural infection and the presence in some cases of mild respiratory illness, dry coughs, and a loss of appetite have been documented (CDC, 2020; Daly, 2020), currently, there is not enough information or official consensus of expected diseases in cats (either domestic or wild) infected with SARS-CoV-2.

There have been multiple discussions about the possible role of cats in SARS-CoV-2 epidemiology, and it has been proposed that the presence of a compatible receptor is enough evidence to state that animals may play roles in transmitting SARS-CoV-2 to humans (Sun et al., 2020). We believe that our results and results from others (Damas et al., 2020; Devaux et al., 2020; Wu et al., 2020) should be a starting point to understand and evaluate the role of SARS-CoV-2 epidemiology in the context of the *One Health* concept (Tilocca et al., 2020). Also, in this context, it is reasonable to suppose that cats may be a silent intermediate host of SARS-CoV-2, due to the fact that infected cats may not show any appreciable symptoms that might be recognized by their owners or signs could be not too specific (CDC, 2020; Halfmann et al., 2020).

Whether future research proves that the virus can transmit between humans and cats, this may cause widespread panic and motivate people to abandon their cats for the fear of infection (Gao et al., 2020). Recent analysis of data from 1000 simulations indicates that fear over domestic cats may be unnecessary, as abandoning domestic cats may cause even more people to be infected overall (Gao et al., 2020) and that possibly the best strategy for controlling the virus spread is to quarantine pets at home (Grimm, 2020). Besides, due to the fact that human-to-human transmission is the driving force of the pandemic, the possible impact of the animal-to-human transmission is small in this pandemic context; on the contrary, we must emphasize efforts on the understanding of the impact of SARS-CoV-2 in animal populations and understand the shared risk for the post-pandemic future.

## Conclusion

Overall, our results suggest that SARS-CoV-2 could efficiently interact with ACE2 orthologs in non-primate species. We showed that the cACE2, tACE2, and fACE2 cellular receptors could potentially interact with RBD of SARS CoV-2 Spike protein and that these receptors share the same virus-binding interface with hACE2. In contrast, dACE2 and haACE2 were not predicted to establish stable interactions with the SARS-CoV-2 S protein RBD, suggesting that these species are less susceptible to the infection. Although susceptibility has been predicted *in silico* and has been shown *in vivo* in cats, further investigations are required to respond to multiple questions about SARS-CoV-2 pathogenesis in this species, as well as their role in viral transmission to humans.

## Data Availability Statement

The original contributions presented in the study are included in the article/supplementary material, further inquiries can be directed to the corresponding authors.

## Author Contributions

SR-M: methodology, investigation, writing - original draft, writing - review and editing, and visualization. MM-G: conceptualization, writing - original draft, and writing - review and editing. GW: writing - review and editing and visualization. JJ: methodology, writing - review and editing, supervision, and visualization. JR-S: conceptualization, writing - original draft, writing - review and editing, supervision, and funding acquisition. All authors contributed to the article and approved the submitted version.

### Conflict of Interest

The authors declare that the research was conducted in the absence of any commercial or financial relationships that could be construed as a potential conflict of interest.
